# LONG-TERM OUTCOMES FOLLOWING A PULMONARY TELEREHABILITATION TRIAL FOR PEOPLE WITH RESPIRATORY POST-ACUTE SEQUELAE OF COVID: A 12-MONTH FOLLOW-UP STUDY

**DOI:** 10.2340/jrm.v58.44828

**Published:** 2026-02-26

**Authors:** Jack M. REEVES, Lissa M. SPENCER, Ling-Ling TSAI, Andrew J. BAILLIE, Joshua BISHOP, Amanda MCANULTY, Yuna HAN, Regina LEUNG, Jennifer A. ALISON

**Affiliations:** 1 Sydney School of Health Sciences, Faculty of Medicine and Health, The University of Sydney, Sydney, NSW; 2 Graduate School of Health, Faculty of Health, The University of Technology Sydney, Sydney, NSW; 3 Physiotherapy Department, Royal Prince Alfred Hospital, Sydney, NSW; 4 Professorial Unit, Allied Health, Sydney Local Health District, Sydney, NSW; 5 Physiotherapy Department, Balmain Hospital, NSW; 6 Physiotherapy Department, Canterbury Hospital, NSW; 7 Physiotherapy Department, Concord Repatriation General Hospital, Sydney, NSW; 8 Department of Thoracic Medicine, Concord Repatriation General Hospital, Sydney, NSW, Australia

**Keywords:** post-acute sequelae of COVID (PASC), respiratory symptoms, telerehabilitation, cohort study

## Abstract

**Objective:**

To determine whether changes in physical and psychological outcomes occurred in the 12 months following completion of a randomized controlled trial comparing a 4-week pulmonary telerehabilitation programme with a control group of usual care in people with respiratory post-acute sequelae of COVID.

**Methods:**

This was a prospective, observational, multi-site, assessor-blinded study. Primary outcome: 1-minute sit-to-stand test. Secondary outcomes: 5-repetition sit-to-stand test; Montreal Cognitive Assessment; COVID-19 Yorkshire Rehabilitation Scale; COPD Assessment Test; 36-Item Short-Form Health Survey; Hospital Anxiety and Depression Scale; Fatigue Severity Scale; and the Kessler Psychological Distress Scale. All outcomes were assessed at baseline and 12 months following randomized controlled trial participation. All participants were analysed as a single group at 12 months, given there were no significant differences in the randomized controlled trial.

**Results:**

Of 50 participants enrolled in the randomized controlled trial, 29 (58%) participated in the 12-month follow-up. Compared with baseline, at the 12-month follow-up there was no statistically significant improvement in the primary outcome of the 1-min sit-to-stand test (1.5 points, CI: –1.3 to 4.2), yet statistically significant differences in the 5-repetition sit-to-stand test (–1.4 seconds CI: –2.7 to –0.1), COPD Assessment Test (–4.1 points CI: –6.8 to –1.4), and some domains of SF-36 and COVID-19 Yorkshire Rehabilitation Scale.

**Conclusion:**

This study demonstrated that people reporting respiratory post-acute sequelae of COVID experienced some recovery at 12 months, despite not improving initially during a 4-week pulmonary telerehabilitation programme or control period.

## LAY ABSTRACT

This study explored the recovery of people with persistent respiratory symptoms following COVID-19 infection who participated in a programme of pulmonary rehabilitation as part of a clinical trial. The clinical trial found no significant differences between those who participated vs did not participate in the pulmonary rehabilitation programme. Despite this, among the participants who were reassessed 12 months following participation in the trial, there were significant improvements in respiratory symptoms, lower-limb functional performance, and fatigue. This study demonstrates that individuals may experience improvement in post-COVID symptoms over time.

Post-acute sequelae of COVID-19 (PASC) are multisystemic and encompass >200 unique symptoms, and while the exact pathogenesis is unknown, current hypotheses include immune dysregulation, blood clotting and endothelial abnormality, dysfunctional neurological signalling, autoimmunity, and microbiota disruption (1). A large systematic review and meta-analysis has shown that 45% of people will have at least one unresolved symptom following infection with COVID-19, regardless of whether they were hospitalized during the acute phase of infection (2). Based on a separate systematic review including patients who were and were not hospitalised at the time of infection, respiratory symptoms are frequently reported following the acute period of infection, with cough affecting 18% of individuals and dyspnoea occurring in 21% (3).

Pulmonary rehabilitation (PR) (4) and pulmonary telerehabilitation (PTR) (5) are both highly effective interventions for improving respiratory symptoms such as dyspnoea, functional capacity, health-related quality-of-life (HRQoL), anxiety, and depression in people with chronic respiratory disease. Given the presence of respiratory symptoms in some individuals experiencing PASC, PR and PTR have been suggested as appropriate interventions to address COVID sequelae (6). Challenges exist in providing rehabilitation services that are effective and evidence-based due to the diverse range of symptoms reported (7). To highlight these challenges, a recent systematic review of 16 randomized controlled trials (RCTs) of telerehabilitation for people with PASC evaluated physical function, HRQoL, symptoms, and psychological function. They found a pooled difference in favour of telerehabilitation over usual care in physical function, but heterogenous effects in all other outcomes and evidence grades were “low” and “very low” for all outcomes (8).

While the most effective forms of rehabilitation remain unknown, most individuals improve over time following COVID-19 infection. A large 2-year follow-up study of 1,192 patients who were discharged from hospital following COVID-19 infection demonstrated statistically significant improvements in multiple physical and psychological outcomes between 2 months and 2 years post hospitalization (9). Regardless, participants still had a high symptom burden at the 2-year timepoint, including 14% who still had a Modified Medical Research Council (mMRC) Dyspnoea Scale score of at least 1, which signifies functional disability due to breathlessness, 30% who had fatigue or muscle weakness, and 25% who had sleep difficulties.

This study assessed the 12-month outcomes of participants of a randomized controlled trial (RCT) that evaluated a 4-week pulmonary telerehabilitation intervention compared with usual care of no rehabilitation for people with respiratory PASC. There were no significant between-group differences in any outcomes at completion of the trial. As such, the aim of this cohort study was to determine whether any changes in physical and/or psychological outcomes occurred in the 12 months following participation in the RCT (10).

## MATERIALS AND METHODS

### Study design and setting

**Fig. 1:**
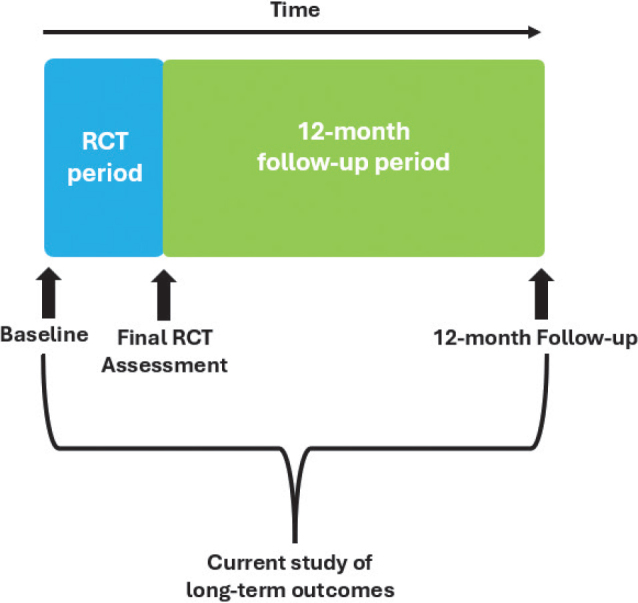
Study timepoints.

This was a prospective, observational, 12-month follow-up cohort study of a subgroup of participants from a multi-site, assessor-blinded RCT. The protocol for the RCT and this nested 12-month follow-up study has been published previously where the methods are described in more detail (11). Briefly, the RCT delivered a 4-week pulmonary telerehabilitation programme of individualized exercise and education in a group format vs a control of usual care, which involved an educational pamphlet on managing symptoms and any required medical follow-up. The RCT protocol allowed participants randomized to the control group (CG) to cross -over into the intervention (PTR) following the control period. There were no significant between-group differences in the any outcome (10). This nested study compared 12-month follow-up with baseline measures (Fig. 1). The study used the Strengthening the Reporting of Observational Studies in Epidemiology (STROBE) Statement guidelines for reporting observational research and was approved by the Sydney Local Health District (SLHD) Human Research and Ethics Committee (Royal Prince Alfred Zone), registered (ACTRN 12622000355774), and funded by a SLHD research grant. It is conducted in accordance with the Declaration of Helsinki. Data of the 12-month follow-up were collected between July 2023 and August 2024.

### Participants

Participants were those with respiratory symptoms (including dyspnoea, cough, or wheeze), as confirmed by a respiratory physician, at least 4 weeks following confirmed diagnosis of SARS-CoV-2 on either a polymerase chain reaction (PCR) test or a rapid antigen test (RAT). Requiring a confirmed diagnosis of SARS-CoV-2 did not impact recruitment because, at this stage of the pandemic in Australia, testing for COVID-19 was free and mandatory for anyone with symptoms. We chose 4 weeks post-infection to exclude the “acute phase” of COVID, which the National Institute for Health and Care Excellence (NICE) defines as within the first 4 weeks from infection.

Prior to recruitment, all participants attended a Post-COVID Respiratory Clinic, where they were screen-ed for inclusion in the study (11). The post-COVID respiratory clinic involved a review by a respiratory physician, lab-based pulmonary function tests, and screening by a physiotherapist for suitability for rehabilitation. Referral criteria included any patient with confirmed SARS-CoV-2 infection (through PCR or RAT) reporting respiratory symptoms at least 6 weeks following infection. Patients were eligible for the clinic regardless of the severity of their initial SARS-CoV-2 infection, i.e., those who were hospitalized or non-hospitalized were included. Patients had to be referred to the clinic by a medical doctor, either a specialist or a general practitioner. During the clinic’s operation there were 260 patients who were referred and attended an initial appointment (12).

The inclusion criteria for the RCT were: (*i*) people with respiratory sequelae who attended a post-COVID respiratory clinic with symptoms of at least 4 weeks’ duration; (*ii*) those identified by their treating physician as suitable for rehabilitation; (*iii*) ≥18 years of age; (*iv*) able to provide informed consent. The exclusion criteria were: (*i*) people with a severe COVID-19 infection admitted to an intensive care unit and who develop post-ICU syndrome; (*ii*) acute symptoms of any illness where exercise is not recommended; (*iii*) medically unstable as diagnosed by their treating physician; (*iv*) pregnant or post-partum women; (*v*) no access to appropriate technology (i.e., internet or computer); (*vi*) difficulty understanding English; and (*vii*) unable to access an interpreter; (*viii*) severe cognitive impairment or other comorbidities that would make remote exercise unsafe as assessed by the physician.

All participants recruited to the RCT were eligible for participation in the 12-month follow-up study and were invited through a phone call followed by an email containing a participant information sheet. In addition to earlier consent, all participants provided verbal informed consent during a videoconference call. A $50 (AUD) voucher was offered as reimbursement for time.

### Outcomes

All outcomes were collected remotely by an experienced physiotherapist with training in pulmonary telerehabilitation. The sit-to-stand and the Montreal Cognitive Assessment Test-Blind version (MoCA-BLIND) were conducted during a videoconference (Zoom Video Communications Inc.) while questionnaires were completed online by participants using secure Research Electronic Data Capture (REDCap; https://project-redcap.org/) data management software. Further details concerning our selected outcomes measures and reasoning behind their selection can be found in our published protocol (11) and RCT report (10).

*Physical outcomes.* The primary outcome was the number of repetitions achieved in the 1-minute sit-to-stand test (1-minSTST), which is a measure of functional capacity (13). The 1-minSTST has a moderate correlation with the 6-minute walk test (6MWT) (14), including in a post-COVID population (15), and has been validated and widely used across the age-span (16). Time to complete the 5-repetition sit-to-stand test (5STST) was also evaluated. The 5STST is a measure of lower limb functional performance and can be safely performed remotely (17). A standard chair of 46 cm height without armrests (or otherwise a chair closest in height if the standard chair was not available) was used to standardize the sit-to-stand tests (13). The same chair was used at all assessment timepoints. Participants rested for 15 min between tests.

*Cognitive outcomes.* The MoCA-BLIND (18) was administered by a physiotherapist remotely for the assessment of cognitive function. As the follow-up was greater than 3 months after the initial MoCA (the boundary for possible practice effects for repetitive use), the same version (Version 7.1) that was used at baseline assessment was also used at the 12-month follow-up assessment.

*Patient-reported outcomes.* The patient-reported outcomes were collected via REDCap. Fatigue was measured using the Fatigue Severity Scale (FSS) (19), which is a 9-item measure of fatigue severity used for patients with a variety of conditions and had been deemed suitable for measuring fatigue in individuals post-COVID (20).

Anxiety and depression was measured using the Hospital Anxiety and Depression Scale (HADS) (21), a 14-item validated questionnaire (22), and psychological distress using the Kessler Psychological Distress Scale (K6+) (23), a validated and reliable questionnaire with 6 items (24). Respiratory symptoms were measured using the Chronic Obstructive Pulmonary Disease Assessment Test (CAT) (25) which, although intended for patients with obstructive respiratory pathology, is widely used for the evaluation of respiratory symptoms in many contexts and in those with chronic conditions.

Participant HRQoL was measured using the 36-Item Short-Form Health Survey (SF-36) (26), a questionnaire that provides a score for 8 health domains. The COVID-19 Yorkshire Rehabilitation Scale (YRS-C19) (27) was also used as a comprehensive questionnaire evaluating COVID-sequelae-related health. The return-to-work questions from the International Severe Acute Respiratory and emerging Infection Consortium (ISARIC) questionnaire (28) were also collected as an exploratory outcome to provide information on changes to work status and work capability in the context of COVID sequelae.

### Statistical analysis

Statistical analysis was performed using IBM SPSS version 29 (IBM Corp, Armonk, NY, USA). Normality was assessed using a Shapiro–Wilk Test. The appropriate statistical test (i.e., paired *t*-test or Wilcoxon signed-rank test) was used to evaluate within-group differences between baseline and 12-month follow-up for all outcomes, with 95% confidence intervals reported for within-group change. An exploratory post-hoc analysis was performed of participant characteristics (age, body mass index [BMI]) of those who met the minimal clinically important difference (MCID) in 1-minSTST, 5STST, CAT, and FSS, and Pearson’s correlation was used for continuous variables to evaluate linearity. We did not analyse categorical variables as the size of the categorial groups was too small for meaningful interpretation. The level of significance for all outcomes was set at an alpha of <0.05.

## Results

Twenty-nine participants of a possible 50 (58%) completed the study. Reasons for not participating in the 12-month follow-up were: not interested (*n* = 4, 19%), too busy to participate (*n* = 4, 19%), unable due to “brain fog” (*n* = 1, 5%), or no reason given as lost to follow-up (*n* = 12, 57%). Of the 29 participants, 17 (59%) had been randomized to PTR, 8 (28%) had been randomized to the CG and crossed over to PTR after the control period, and 4 (14%) had been randomized to the CG and chose not to cross over. The time from the initial baseline assessment to final 12-month follow-up assessment was 15±1 months, as this included the time of the intervention or control periods and was approximately 12 months after the final assessment of the RCT (see Fig. 1). Characteristics of those who completed the 12-month follow-up in comparison with all participants in the RCT are presented in Table I.

**Table I:** Characteristics of participants who completed the 12-month follow-up in comparison with all participants in the randomized controlled trial (RCT)

Variables	12-month follow-up (*n* = 29)	RCT Participants (*n* = 50)
Age, years, mean ± SD	53 ± 11	54 ± 14
Female, *n* (%)	17 (59%)	30 (60%)
BMI, kg/m^2^, mean ± SD	31 ± 9	30 ± 8
Hospitalized, *n* (%)	5 (17%)	10 (20%)
Smoking history, *n* (%)	14 (48%)	24 (48%)
History of respiratory disease, *n* (%)	12 (41%)	17 (34%)
Time since infection, months, mean ± SD	20 ± 6	NA

SD: standard deviation; BMI: body mass index; NA: not applicable. Respiratory disease includes chronic obstructive pulmonary disease, interstitial lung disease, bronchiectasis, or asthma.

Outcomes at baseline and 12-month follow-up are given in Table II. Compared with baseline, at the 12-month follow-up there was no statistically significant improvement in the primary outcome of the 1-minSTST (1.5 points, CI: –1.3 to 4.2) (absolute value at 12 months 24.3±8.1 repetitions; compared with the normative value of approximately 33 based on the mean age of cohort [29]). However, there were statistically significant improvements in the 5STST (–1.4 s, CI: –2.7 to –0.1) (absolute value at 12 months 11.0±4.0 s; compared with the normative value 7.8±2.8 s [30]) and the CAT score (–4.1 points, CI: –6.8 to –1.4) (absolute value at 12 months 15.0±7.3; compared with normative value of <10 [31]). To understand what specific respiratory symptoms improved, change in score for each question in the CAT was evaluated (Table III). There were statistically significant changes in the questions “I am not limited doing any activities at home”, “I am confident leaving my home despite my lung condition”, and “I have lots of energy”, with a lower score indicating improvement.

**Table II:** Differences between baseline and 12-month follow-up (*n* = 29)

Factor	Baseline Mean ± SD	12 months post-intervention Mean ± SD	Within-group Mean difference (95%CI)	*p*-value
1-minSTST, repetitions	22.9±6.3	24.3±8.1	1.5 (–1.3 to 4.2)	0.277
5STST, s	12.4±3.3	11.0±4.0	–1.4 (–2.7 to –0.1)	**0.045**
CAT, score	19.1±6.6	15.0±7.3	–4.1 (–6.8 to –1.4)	**0.004**
HADS, score				
Anxiety	8.9±3.7	8.4±4.4	–0.5 (–2.0 to 1.1)	0.527
Depression	7.9±4.4	7.1±4.5	–0.8 (–2.2 to 0.7)	0.287
FSS, score	49.4±12.3	44.0±11.9	–5.4 (–11.1 to 0.3)	0.063
K6+, score	9.4±4.2	8.5±5.6	–1.0 (–2.5 to 0.6)	0.220
MoCA-Blind, score	19.7±2.3	19.2±2.1	–0.5 (–1.5 to 0.6)	0.349
C19-YRS, score				
Breathless – rest	2.1±2.3	1.6±2.2	–0.5 (–1.2 to 0.2)	0.185
Breathless – dressing	3.0±2.4	2.3±2.6	–0.7 (–1.7 to 0.3)	0.159
Breathless – stairs	5.9±2.5	4.4±2.9	–1.4 (–2.4 to –0.4)	**0.007**
Mobility	3.2±2.6	3.2±3.0	–0.1 (–0.8 to 0.7)	0.921
Fatigue	6.8±2.3	5.0±3.1	–1.8 (–3.1 to –0.6)	**0.006**
Pain/discomfort	4.0±2.5	1.8±2.3	–2.2 (–3.4 to –1.0)	**< 0.001**
Anxiety	4.8±2.8	4.1±2.6	–0.8 (–1.6 to 0.10	0.087
Depression	5.5±7.3	3.2±3.1	–2.2 (–5.1 to –1.0)	0.124
Symptoms severity subscale	26.4±13.1	22.2±15.6	–4.2 (–8.5 to 0.2)	0.059
Functional disability subscale	14.8±7.5	11.8±9.9	–3.0 (–6.3 to 0.3)	0.070
Global perceived health	4.6±1.8	6.5±1.8	1.9 (0.7 to 3.00	**0.003**
SF-36, score				
Physical functioning	36.4±19.2	52.6±26.7	16.2 (6.5 to 25.9)	**0.002**
Role, physical health	19.4±32.8	25.3±38.0	5.9 (–11.7 to 23.4)	0.498
Role, emotional problems	32.0±44.6	45.3±42.9	13.3 (–4.0 to 30.6)	0.125
Energy/fatigue	45.2±7.5	36.9±22.7	–8.4 (–18.9 to 2.1)	0.113
Emotional well-being	50.5±13.3	62.2±21.9	11.7 (4.5 to 18.8)	**0.003**
Social functioning	48.0±23.8	55.0±28.6	7.0 (1.3 to 4.4)	0.219
Pain	58.2±22.8	59.1±29.0	1.0 (–8.1 to 10.1)	0.830
General health	39.4±17.6	40.4±23.0	1.0 (–6.0 to 8.0)	0.778

SD: standard deviation; CI: confidence interval; 1-minSTST: 1-minute sit-to-stand test; 5STST: 5 repetition sit-to-stand test; CAT: COPD Assessment Test; C19-YRS: COVID-19 Yorkshire Rehabilitation Scale; FSS: Fatigue Severity Scale; HADS: Hospital Anxiety and Depression Scale; K6+: Kessler Psychological Distress Scale; MoCA-Blind: telephone version of the Montreal Cognitive Assessment; SF-36: 36-Item Short-Form Health Survey. Statistical significance *p*<0.05 (highlighted in bold).

**Table III:** Within-group changes for CAT questions

CAT question	Baseline Mean ± SD	12 months post intervention Mean ± SD	Within-group Mean difference (95%CI)	*p*-value
Q1 – Cough	1.9 ± 1.5	1.5 ± 1.4	–0.4 (–0.8 to 0.1)	0.115
Q2 – Sputum	0.9 ± 1.1	1.0 ± 1.4	0.1 (–0.3 to 0.5)	0.600
Q3 – Chest tightness	2.0 ± 1.3	1.6 ± 1.3	–0.4 (–1.1 to 0.2)	0.161
Q4 – Breathlessness with hills and stairs	3.7 ± 1.3	3.1 ± 1.3	–0.5 (–1.1 to 0.2)	0.060
Q5 – Activities of daily living	3.0 ± 1.4	1.6 ± 1.5	–0.7 (–1.3 to –0.2)	**0.015**
Q6 – Confidence leaving the home	1.6 ± 1.5	0.9 ± 1.2	–0.7 (–1.3 to –0.2)	**0.011**
Q7 – Sleep	2.5 ± 1.5	2.1 ± 1.3	–0.4 (–1.1 to 0.2)	0.177
Q8 – Energy	3.7 ± 1.0	2.6 ± 1.4	–1.1 (–1.7 to –0.4)	**0.003**

SD: standard deviation; CAT: COPD Assessment Test; CI: confidence interval. Statistical significance *p*<0.05 (highlighted in bold).

In the YRS-C19 there were statistically significant improvements in ratings of breathlessness on stairs, fatigue in comparison with pre-COVID illness, and severity of pain or discomfort. In the SF-36, there were statistically significant improvements in the domains of “physical functioning” and “emotional well-being”.

**Fig. 2:**
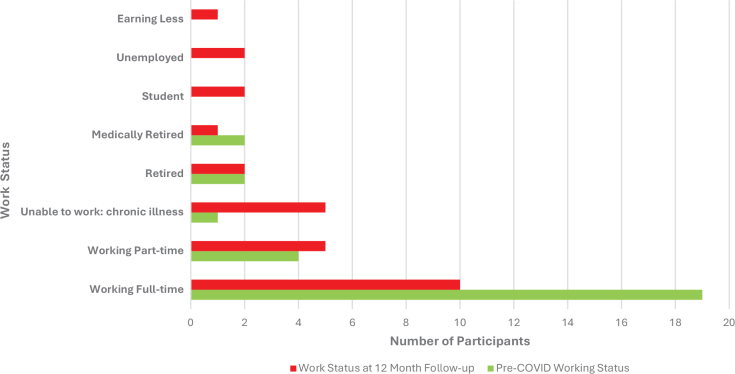
Work status outcomes from the ISARIC COVID Survey (*n* = 28).

The ISARIC return to work survey was completed by 28 (97%) participants. Fig. 2 demonstrates the number and distribution of the work status of participants before COVID infection and at the 12-month follow-up time point. Of the 28 participants assessed, 15 (54%) reported a change to their work status including: change due to poor health (*n* = 11), being made redundant (*n* = 2), sick leave (*n* = 1), and hours reduced by their employer (*n* = 1). In addition, 17 (61%) reported that at some stage prior to the follow-up assessment they had been away from work due to COVID-19 illness.

Four outcomes with published MCIDs were evaluated against age and BMI (Table IV). These were the 1-minSTST using an MCID of 3 repetitions (32), the 5STST using an MCID of 1.7 s (33), the CAT score using an MCID of 2 points (34), and the FSS using an MCID of 0.45 points (35). Pearson’s correlation of age and BMI with these 4 outcomes are listed in. There was a moderate–strong correlation between BMI and change in FSS scores (*r* = 0.70, *p* = <0.001) (Table V) (36). Those who improved by at least the MCID for the FSS had a lower BMI of 27±5 kg/m^2^ compared with those who did not meet the MCID who had a BMI of 36±11 kg/m^2^. There was no correlation between age and change in any of the 4 outcomes assessed.

**Table IV:** Age and BMI of those who improved by at least the MCID for the 1-minSTST, 5STST, CAT, and FSS

Outcomes	MCID used (Reference)	Age, years		BMI, kg/m^2^	
		MCID met Mean ± SD	Not met Mean ± SD	MCID met Mean ± SD	Not met Mean ± SD
1-minSTST^a^ (*n* = 27)	3 repetitions (Che et al.) (32)	48 ± 9	54 ± 12	27 ± 7	35 ± 9
5STST^b^ (*n* = 25)	1.7 seconds (Jones et al.) (33)	46 ± 18	51 ± 13	30 ± 8	33 ± 10
CAT^c^ (*n* = 27)	2 points (Kon et al.) (34)	52 ± 11	53 ± 12	30 ± 8	36 ± 11
FSS^d^ (*n* = 27)	4.05 points (Rooney et al.) (35)	51 ± 12	55 ± 12	27 ± 5	34 ± 10

SD: standard deviation; MCID: minimum clinically important difference; 1-minSTST: 1-minute sit-to-stand test; 5STST: 5 repetition sit-to-stand test; BMI: body mass index; CAT: COPD Assessment Test; FSS: Fatigue Severity Scale; SF-36: 36-Item. Letters denote number of participants who met the MCID: a = 11; b = 6; c = 17; d = 15.

**Table V:** Pearson’s correlation of age and BMI with change in outcomes (*n* = 29)

Outcomes	Age		BMI	
	Pearson’s *r*	*p*-value	Pearson’s *r*	*p*-value
1-minSTST	–0.21	0.294	–0.32	0.105
5STST	0.10	0.622	0.38	0.059
CAT	–0.04	0.858	0.29	0.146
FSS	–0.01	0.958	**0.70**	**<0.001**

1-minSTST: 1-minute sit-to-stand test; 5STST: 5 repetition sit-to-stand test; BMI: body mass index; CAT: COPD Assessment Test; FSS: Fatigue Severity Scale; SF-36: 36-Item; statistical significance *p*<0.05 (highlighted in bold).

## DISCUSSION

This study reports the changes from baseline to 12 months after completion of an RCT comparing 4-weeks’ PTR to usual care for people with respiratory PASC. The RCT showed no statistically significant between-groups differences for any outcome (10). This prospective 12-month follow-up cohort study included participants in the initial PTR group, those in the CG, and those who crossed over into the PTR group after completing the control period. The study did not demonstrate significant improvement in the 1-minSTST, but did demonstrate statistically significant improvements in the 5STST and the CAT, as well as in some questions from the YRS-C19 and domains of the SF-36, at 12 months compared w baseline. This suggests that people reporting respiratory PASC are likely to experience some recovery over time.

Whilst there was a within-group change of 1.5 repetitions in the 1-minSTST, which was the primary outcome, this change was not statistically significant, nor did it meet the MCID of 3 repetitions. The 5STST, a secondary outcome measuring lower limb functional performance, demonstrated significant within-group change from baseline to 12 -months of –1.4 s. This suggests that, over time, people reporting respiratory symptoms after COVID may improve their knee extension strength (37) potentially resulting in greater ability to climb stairs, rise from a chair, and walk at a desired pace. This finding is supported by evidence of the improvement in the 5STST over a 12-week period in a study population of previously hospitalized COVID-19 patients who did not undergo any rehabilitation (38).

Comparison of baseline with 12-month follow-up data also demonstrated significant improvements in “energy” in the CAT score and reductions in “fatigue’ in the YRS-C19, but did not quite reach significance in the FSS. Fatigue, or lack of energy, is a common persisting symptom, occurring in up to 25% of people following acute COVID-19 infection (39) and is often severe and debilitating (20). Longitudinal data suggest that about half of those reporting persisting fatigue will recover within 2 years (40). Despite no significant improvement in fatigue in either the PTR or CG in the initial RCT, some evidence of improvement in fatigue over 12 months was demonstrated in the current study population, supporting previous evidence of recovery over time in this outcome.

While this study demonstrated evidence of recovery in various symptoms and functional outcomes, the ISARIC return to work survey completed at the 12-month timepoint showed poorer work status of participants in comparison with pre-COVID infection. This was despite improving pandemic-related unemployment rates over the same period (41) and aligns with recent evidence that suggests people with “long COVID” have a higher rate of unemployment, and if working are less likely to be working full time (42). Unemployment can have serious indirect effects on health, with 1 study projecting the long-term effects of COVID-related unemployment to translate to an increase in mortality of 800,000 individuals in the United States in the next 15 years (43). Social interventions that seek to support employment during recovery from COVID-related disability are therefore critical in protecting those with long COVID from further morbidity and mortality.

Another study finding was that people with a higher BMI were less likely to reach the MCID for the FSS, 5STST, and the 1-minSTST over a 12-month period. A recent large systematic review and meta-analysis of global studies (*n* = 13,368,074) evaluated the prevalence of persistent symptoms following COVID-19 infection and demonstrated BMI as a risk factor for having persistent COVID symptoms (39). Our study supported this by showing that BMI may also be a risk factor for delayed improvement in people with respiratory PASC. Another risk factor identified from the meta-analysis was older age; however, there was no correlation between age and change in outcomes in our analysis.

### Limitations

There are several limitations in our study. A between-groups analysis of PTR compared with CG of usual care was unable to be undertaken at the 12-month timepoint due to the option for participants to cross over from the CG to the PTR group following the control period. The option to cross over to PTR was due to advice from a long-COVID lived experience group who felt that all participants needed the opportunity to participate in any rehabilitation being offered as there was very limited access to such interventions at the time of the initial RCT. In addition, due to the novelty of respiratory PASC, there were limited data on rehabilitation outcomes and therefore having a larger group enrolled in the 4-week PTR programme was considered important to increase our knowledge regarding the effects. As the RCT did not show any improvement in outcomes from PTR (10), we were able to consider the group as a whole and complete a within-group comparison of all participants from baseline to 12 months post the completion of the RCT. Another limitation was that only 29 (58%) participants completed the 12-month follow-up assessment, which could represent a potential source of attrition bias if those who did not participate were likely to have poorer outcomes over 12 months. However, only 1 participant cited negative symptoms as a reason for not participating, and the characteristics of those who participated in the 12-month follow-up compared with all participants of the RCT were similar (see Table I). At the time of data collection, we did not collect detail on the type of respiratory disease, only whether the patient had a respiratory disease (yes/no; see Table 1), and therefore the specific type of chronic respiratory disease is not captured. Finally, we are unsure whether participants undertook additional interventions during the 12-month follow-up period. This presents a possible confounder to the improvements seen; however, this is unlikely as there are few known effective interventions for this cohort.

### Conclusion

This study of outcomes 12 months following completion of an RCT in people with respiratory PASC showed statistically significant recovery over time in lower limb functional performance on the 5STST; respiratory symptoms on the CAT score; improvements in the YRS-C19 ratings of breathlessness on stairs, fatigue, and severity of pain and discomfort; and improvements in the SF36 domains of “physical functioning” and “emotional well-being”. Importantly, the study demonstrated that people with physician-diagnosed respiratory PASC experienced recovery over time, despite no improvements following an initial short PTR programme or control period. This knowledge may be reassuring for those presenting with respiratory PASC, regardless of whether they have participated in rehabilitation.
